# Self-assembled raccoon dog parvovirus VP2 protein confers immunity against RDPV disease in raccoon dogs: in vitro and in vivo studies

**DOI:** 10.1186/s12985-021-01549-5

**Published:** 2021-04-15

**Authors:** Linya Xia, Guoliang Luo, Mingjie Wu, Lei Wang, Ning Zhang, Congmei Wu, Yuhe Yin

**Affiliations:** 1 School of Life Sciences, Changchun University of Technology, Changchun, 130012 China; 2 Institute of Special Animal and Plant Sciences of CAAS, Changchun, 130012 China

**Keywords:** Raccoon dog parvovirus, Virus like particle, Trigger factor 16, *Escherichia coli*, Immunogenicity

## Abstract

**Background:**

Raccoon dog parvovirus (RDPV) causes acute infectious diseases in raccoon dogs and may cause death in severe cases. The current treatment strategy relies on the extensive usage of classical inactivated vaccine which is marred by large doses, short immunization cycles and safety concerns.

**Methods:**

The present study aimed at optimization of RDPV VP2 gene, subcloning the gene into plasmid pET30a, and its subsequent transfer to *Escherichia coli* with trigger factor 16 for co-expression. The protein thus expressed was purified with ammonium sulfate precipitation, hydrophobic chromatography, and endotoxin extraction procedures. VLPs were examined by transmission electron microscopy, dynamic light scattering, and the efficacy of VLPs vaccine was tested in vivo.

**Results:**

Results indicated that RDPV VP2 protein could be expressed soluble. Transmission electron microscopy and dynamic light scattering results indicated that RDPV VP2 self-assembled into VLPs. Hemagglutination inhibition antibody titers elicited by Al(OH)_3_ adjuvanted RDPV VLPs were comparable with RDPV inactivated vaccines, and the viral loads in the blood of the struck raccoon dogs were greatly reduced. Hematoxylin and eosin and Immunohistochemical results indicated that RDPV VLPs vaccine could protect raccoon dogs against RDPV infections.

**Conclusions:**

These results suggest that RDPV VLPs can become a potential vaccine candidate for RDPV therapy.

## Introduction

Raccoon dog parvovirus (RDPV) is responsible for causing an acute, highly contagious, infectious viral disease, which may lead to fatal hemorrhagic enteritis in raccoon dogs of all ages [24, 30]. Inactivated virus vaccines have been the mainstay of the classical vaccine strategy for RDPV, which despite their higher efficacy suffer from the limitation of incomplete inactivation, thus posing a challenge in vaccine development [1]. The amount of formaldehyde used in the production process of inactivated vaccines often exceeds the safety standards causing concerns during use. This is further complicated by the fact that at present there is no parvovirus enteritis vaccine dedicated to raccoon dogs in the market. Therefore, it is imperative to develop a safe alternative vaccine dedicated to this disease [7].

RDPV is an icosahedral non-enveloped virus belonging to the Parvoviridae family. It consists of a single strand of 20–25 nm DNA, two non-capsid proteins NS1, NS2, and three capsid proteins VP1, VP2 and VP3 [27]. The VP2 protein is found amongst all of the immunogenic epitopes and is capable of generating specific antibody response [12].

Virus-like particles (VLPs) spontaneously assembled by VP2 protein can mimic the three-dimensional structure of natural viruses. VLPs determine the type of antigen and host range, and can stimulate the immune response mediated by B cells. NS gene is known to be essential for replication, and VP gene encodes various forms of the structural protein. As the main capsid protein of the virus, VP2 can self-assemble into VLPs. [5, 9, 15].

The trigger factor (Tf) is a protein with a size of about 50 ku, which is an important member of the *Escherichia coli* molecular chaperones. Tf transiently attaches to a point on the ribosome, forming a protective area and restricting the access of proteases and other downstream factors to the nascent polypeptide chain. This helps in preventing the newly synthesized polypeptide chain from aggregating during folding [10]. Previous studies have shown that several VP2 proteins could be co-expressed in soluble form with Tf using the Prokaryotic expression system [15, 26, 28].

In the present work, we studied the co-expression of VP2 protein and Tf16 using recombinant plasmid pET30 in *E. coli*. A novel attempt was made to purify the RDPV VP2 protein and its self-assembly into VLPs was also studied. Further, the immune efficacy of RDPV VLPs vaccine was evaluated in vivo.

## Materials and methods

### Virus and cells

Feline kidney 81 (F81) cell line was grown in DMEM medium (Gibco, America) containing 10% fetal bovine serum (FBS) (HyClone, America), 100 mg/mL of streptomycin, 100 U/mL of penicillin in T75 flasks (Corning, America) at 37 °C in an atmosphere of 5% CO_2_ and humidified air. At 60% confluency, RDPV RPSN (Chinese Academy of Agricultural Sciences) was added to the T75 flasks. After incubation for 2 h at 37 °C, the medium was changed to DMEM containing 3% FBS. The cells were then cultured continuously for about 48 h. At 80% confluency, the infected cells exhibited cytopathic effects (CPE). The T75 flasks were exposed to a freeze/thaw cycle at −20 °C to recover the virus. The virus was kept at −80 °C, and RDPV RPSN was used for all immunological tests.

### Plasmid construction

For optimization of expression of RDPV VP2 protein, the sequence of RDPV VP2 was optimized based on *E. coli*. The complete RDPV VP2 gene was restriction digested by HindIII, NdeI (TaKaRa, China), and cloned into pET30a (pET30a-VP2) by T4 DNA ligase (TaKaRa, China).

### RDPV VP2 protein expression and co-expression

The recombinant plasmid pET30a-VP2 was transferred to competent *E. coli* ER2566 cells by heat shock method. The positive colonies were incubated in LB medium supplemented with 50 µg/mL kanamycin, 0.2 mmol/L isopropyl β-D-thiogalactoside (IPTG). The recombinant plasmid pET30a-VP2 and Tf16 were then transformed into competent *E. coli* ER2566 cells (TaKaRa, China) by heat shock method. The positive colonies were incubated in LB medium supplemented with 50 µg/mL kanamycin, 20 µg/mL chloramphenicol, 2 mg/mL L-Arabinose and 0.2 mmol/L IPTG. After induction with IPTG at 25 °C for 16 h, the cells were collected and lysed by sonication system in buffer containing 50 mM Tris, 250 mM NaCl (pH 8.0) at 4 °C. The homogenate was centrifugated at 10,000*g* at 4 °C for 30 min. The supernatant and debris were collected and analyzed.

### Purification of RDPV VP2 protein

The collected supernatant was purified by ammonium sulfate precipitation followed by Capto Butyl ImpRes hydrophobic chromatography (GE, USA). The chromatography column was washed using a buffer containing 200 mM (NH_4_)_2_SO_4_, 20 mM Tris, 2 mM NaCl until the UV spectra had no significant changes by NGC (Bio-Rad, America). RDPV VP2 protein was washed in buffer containing 200 mM (NH_4_)_2_SO_4_, 20 mM Tris, 2 mM NaCl and analyzed by SDS-PAGE. Following purification with Triton X-114 (Solarbio, China) extraction, the concentration of endotoxin in the purified RDPV-VP2 protein was measured by Limulus lysate gelatin assay kit (CRL, America). Briefly, after adding a final concentration of 1% of Triton X-114 to RDPV VP2 protein, the mixture was incubated and stirred continuously on ice for 30 min. Then, the mixture was incubated and stirred continuously at 37 °C for 15 min. After centrifugation at 8000 g at 25 °C for 30 min, the RDPV VP2 protein and Triton X-114 were separated. The RDPV VP2 protein so obtained was subjected to another 2 cycles of treatment.

### RDPV VLPs self-assembly and characterization

RDPV VP2 protein was incubated with different concentrations of buffer containing NaCl (150 mM, 250 mM, 500 mM) and at different pH (pH 7.0 and 8.0). The collected RDPV VLPs were determined by DLS, TEM, hemagglutination (HA) assay.

### Raccoon dog immunization with RDPV VLPs

Twenty-five raccoon dogs were divided into 5 groups (n = 5), and were immunized by intramuscular injection. Groups A, B, C used 10 μg, 50 μg, and 100 μg RDPV VLP treated with 20 mg/ml Al(OH)_3_ (Thermo, USA), respectively. In addition, group D were vaccinated with 100 μL of experimentally inactivated RDPV vaccine (HA titer 1:2^11^). Group E was vaccinated with 100 μL PBS. Blood sample was obtained from the veins of the forelimb at 14, 28, 42, 56, 70, 84, 98 days post-inoculation (dpi). The blood samples were centrifuged at 4000 rpm/min for 15 min. The extracted serum was inactivated at 56 °C for 30 min. RDPV RPSN virus was used as antigen (HA titer 1:2^4^). The mixtures were incubated with 1.0% pig erythrocytes in a 96-well V-shaped microplates for Hemagglutination inhibition. The raccoon dogs immunized for 14 days were euthanized, the injection site was observed grossly, and evaluation of the cadavers was done to assess the safety of this vaccine.

### Changes in the physiological parameters of immunized raccoon dogs upon virus challenge

Twenty-five raccoon dogs were divided into 5 groups (n = 5). Groups A, B, C were immunized with either 10 μg, 50 μg, or 100 μg RDPV VLP treated with 20 mg/mL Al(OH)_3_ gel. Additionally, group D was vaccinated with 100 μL of experimentally inactivated RDPV vaccine (HA titer 1:2^11^), while group E was vaccinated with 100 μL of PBS. Raccoon dogs described above were struck with RDPV RPSN virus (HA titer 1:2^11^) containing 10 mL oral MEM medium (Gibco, USA) at 21 dpi. The diet, mental health and the stool samples of raccoon dogs were evaluated. Stool samples were collected and tested with canine parvovirus detection kit (Sinohp, China). Additionally, 1 g stool samples were mixed evenly with 1 mL PBS, and the mixture was centrifuged at 10,000*g*/min for 5 min. The HA titer was estimated from the collected supernatant. Blood samples were collected and analyzed with a Blood Chemistry Analyzer (ABAXIS, USA). The extracted serum was used to detect HI titer. Stool and blood samples from every group were collected and labelled at 8 o'clock every morning.

### Pathological evaluation of raccoon dogs post-inoculation

Pathological examination was carried out for the raccoon dogs at 11 dpi. Various organs i.e. heart, liver, spleen, lungs, kidneys and intestinal tract were collected for examination. The specified organs were removed and stored in 10% formalin, embedded in paraffin, sectioned and stained with hematoxylin and eosin (H&E). Immunohistochemical (IHC) examination was performed using rabbit anti-RPSN polyclonal antibody (anti-RPSN pAb) to evaluate paraffin-embedded sections. Target area (×200) of the tissue was selected by Eclipse Ci-L microscope camera. The positive cumulative optical density value of each field of view was marked as A, while the area of the corresponding tissue pixel was measured and recorded as B. The areal density (C) was calculated as: A/B.

### Ethics approval and consent to participate

All animal experiments were approved by Animal Experiment Committee of Specialty Products Research Institute of the Academy of Agricultural Sciences. The protocols were performed in accordance with the guidelines for the Welfare and Ethics of Laboratory Animals of China.

## Results

### RDPV VP2 protein expression and co-expression

**Fig. 1:**
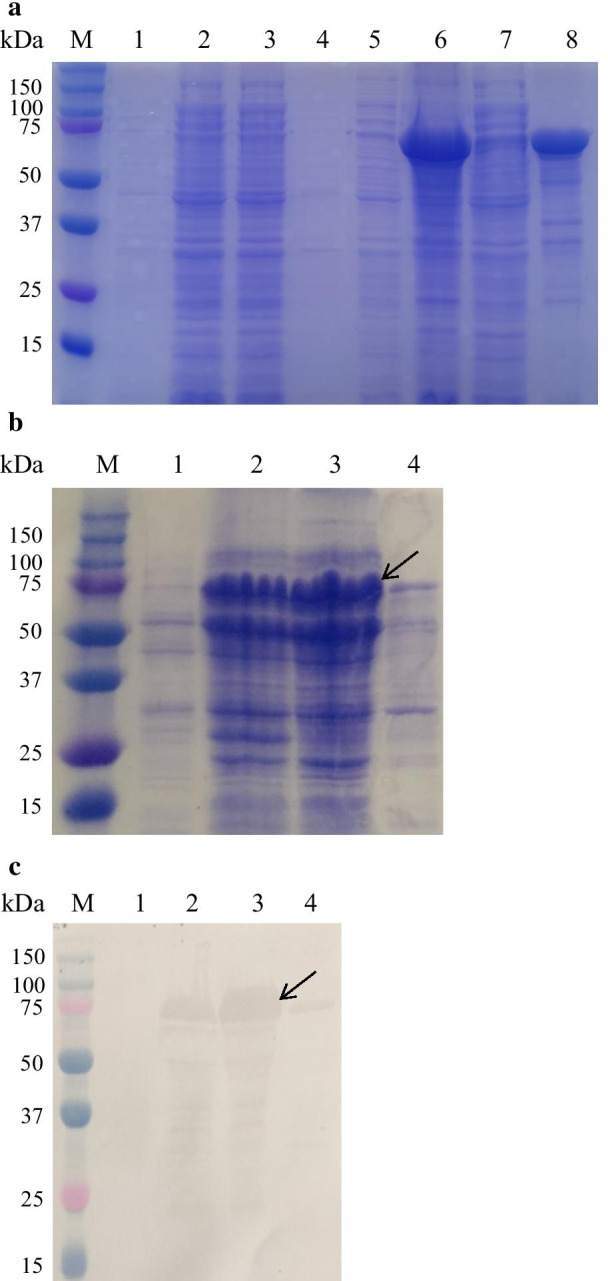
Expression and co-expression of RDPV VP2 protein with Tf16 in *E. coli* ER2566. **a** SDS-PAGE analysis of the expression of VP2 protein in *E. coli* ER2566. Lane M, protein marker; Lane 1, pET30a cells before induced; Lane 2, induced pET30a cells; Lane 3, induced pET30a cells lysate; Lane 4, inclusions of induced pET30a cells; Lane 5, pET30a-VP2 cells before induced; Lane 6, induced pET30a-VP2 cells; Lane 7, induced pET30a-VP2 cells lysate; Lane 8, inclusions of induced pET30a-VP2 cells. **b** SDS-PAGE analysis of the expression of RDPV VP2 protein co-expressed with Tf16. Lane M, protein marker; Lane 1, pET30a-VP2-Tf16 cells before induced of IPTG; Lane 2, induced pET30a-VP2-Tf16 cells; Lane 3, induced pET30a-VP2-Tf16 cells lysate; Lane 4, inclusions of induced pET30a-VP2-Tf16 cells. **c** Western blot analysis of the expression of RDPV VP2 protein co-expressed with chaperone Tf16. Lane M, protein marker; Lane 1, pET30a-VP2-Tf16 cells before induced of IPTG; Lane 2, induced pET30a-VP2-Tf16 cells; Lane 3, induced pET30a-VP2-Tf16 cells lysate; Lane 4, inclusions of induced pET30a-VP2-Tf16 cells

Results showed that a 65 kDa VP2 protein was expressed within the inclusion bodies of induced pET30a-VP2 cells (Fig. 1a). The pET30a-VP2 and Tf16 were transferred to *E. coli* ER2566, where the soluble VP2 protein and protein Tf16 (56 kDa), were co-expressed (Fig. 1b). Western blot analysis of RDPV VP2 expression confirmed that the mouse anti-CPV antibody (Sinohp, China) could bind the target RDPV VP2 protein (Fig. 1c).

### Purification of RDPV VP2 protein

**Fig. 2:**
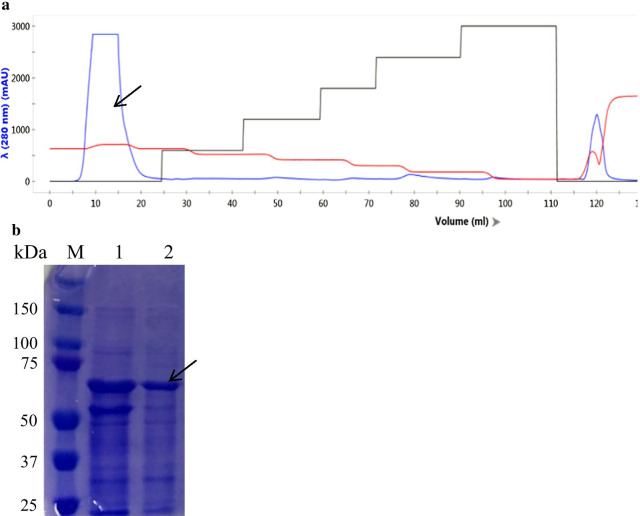
Purification of recombinant proteins RDPV VP2 by hydrophobic chromatography. **a** Hydrophobic chromatography analysis of recombinant proteins RDPV VP2. Black line: %B; Blue line: λ (280 nm); Red line: Conductivity. **b** SDS-PAGE analysis of recombinant proteins RDPV VP2 purified by hydrophobic chromatography. Lane M, protein marker; Lane 1, sample by 30% ammonium sulfate precipitation; Lane 2, purified RDPV VP2 protein

Upon purification by ammonium sulfate precipitation and hydrophobic chromatography, the Tf16 protein (56 kDa) could be removed, and the RDPV VP2 protein (65 kDa) had a purity of 90% (Fig. 2b). This was followed by 3 cycles of Triton X-114 extraction, which showed the concentration of endotoxin in the purified RDPV VP2 protein to be less than 125 EU/mL as estimated by limulus lysate gelatin test kit (Table 1; Fig. 2c) (Charles River, USA). The recovery of RDPV VP2 protein was approximately 72%. Through the BCA protein determination kit (Solarbio, China), the total yield of purified RDPV VP2 protein was estimated to be about 6 g per liter of bacteria, and the recovered protein was 18 mg, which is higher than the previously described protocol by Ji et al. [15].

**Table 1:** Diminution of endotoxin level in the purified RDPV VP2 protein

Program	Sample before chromatography	Sample after chromatography	1 Cycle of Triton X-114	2 Cycles of Triton X-114	3 Cycles of Triton X-114
Endotoxin level (EU/mL)	< 1.25*10^7^	< 1.25*10^5^	< 1.25*10^4^	< 1.25*10^3^	< 1.25*10^2^

### Self-assembly condition of RDPV VLPs

**Fig. 3:**
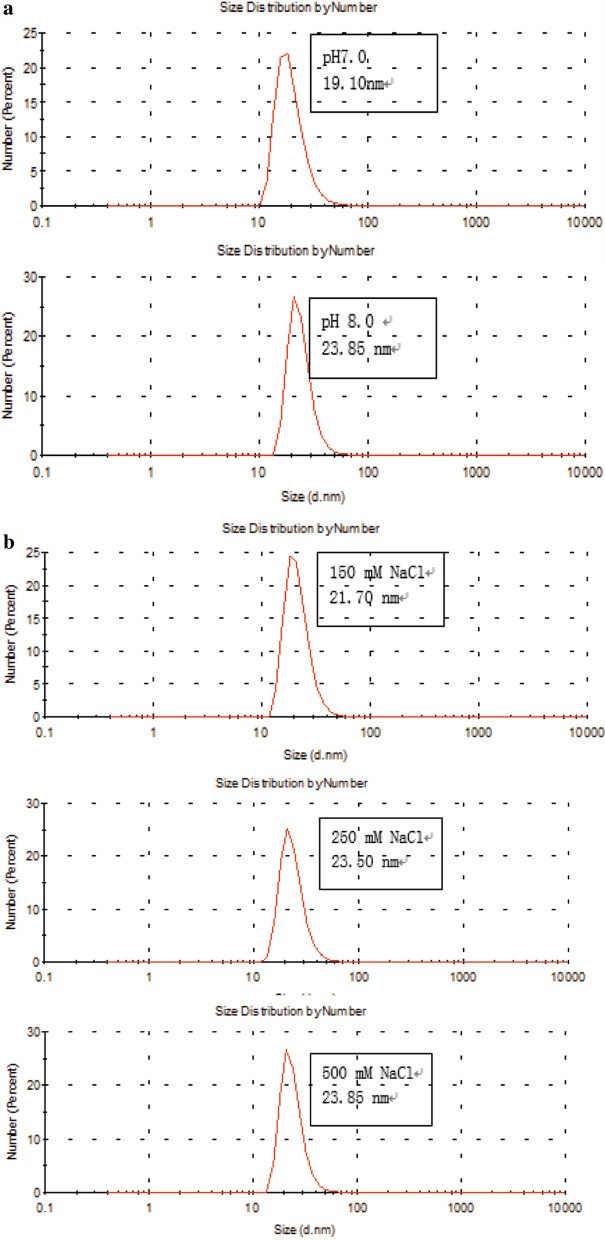

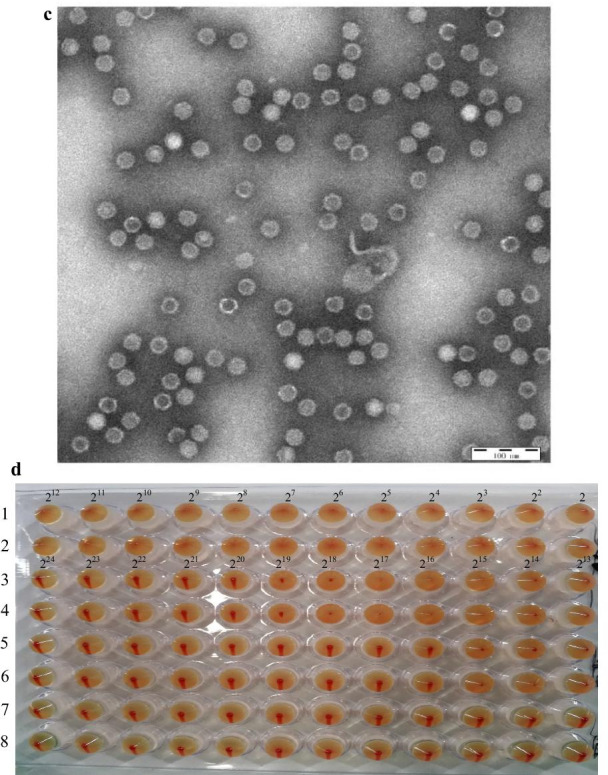
Self-assembly and characterization of RDPV VLPs under heterogeneous conditions. **a** DLS results of RDPV VLPs assembled in pH 7.0 and pH 8.0. **b** DLS results of RDPV VLPs assembled in 150 mM, 250 mM, 500 mM of NaCl at pH 8.0. **c**, Negative staining electron microscopy of RDPV VLPs formed in 250 mM of NaCl at pH 8.0, bar size, 100 nm. **d**, HA of the RDPV VP2 protein. 1–4, the purified RDPV VP2 protein; 5 ~ 6, positive control of RPSN virus; 7 ~ 8, negative control of PBS

DLS results indicated that the size of RDPV VLPs was about 19.10 nm at pH 7.0 and 23.85 nm at pH 8.0 (Fig. 3a). This implied that pH seemed to play a decisive role in the assembly of virus-like particles. The optimal concentration of NaCl was also analyzed at pH 8.0. Results indicated that the size of RDPV VLPs did not change significantly with the increasing concentration of NaCl (Fig. 3b). TEM result showed that purified RDPV VP2 protein could self-assemble into the particles at pH 8.0 and 250 mM NaCl, with a dimension of approximately 24 nm (Fig. 3c). HA assay showed a high HA titer of RDPV VLPs (1:2^18^).

### Evaluation of Immune response to RDPV VLPs in raccoon dogs

**Fig. 4:**
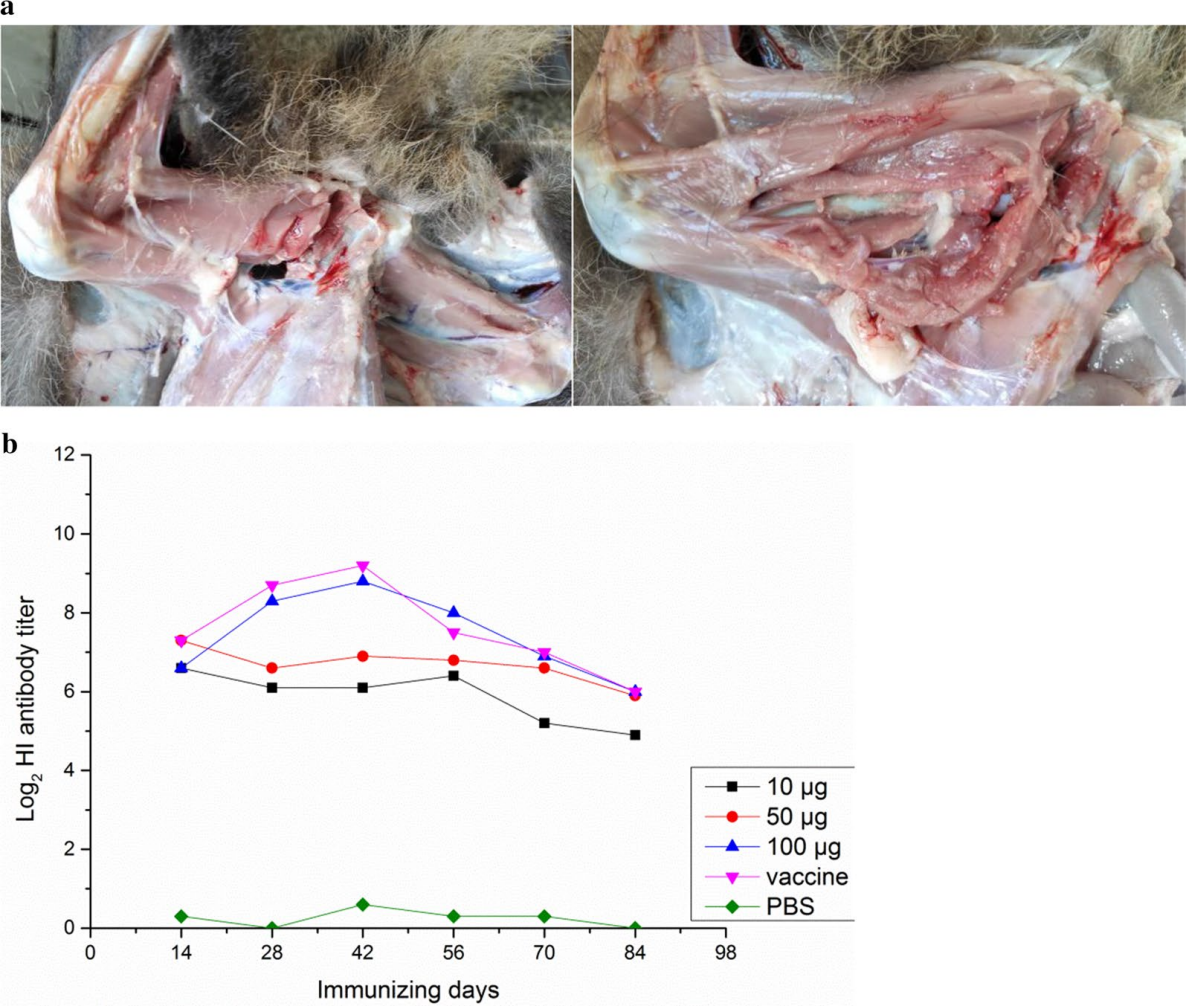
Safety and immunogenicity assay of RDPV VLPs. Groups of raccoon dogs (n = 5) were intramuscularly immunized with 10 µg, 50 µg, 100 µg VLPs treated with Al(OH)_3_, inactivated RDPV vaccine, PBS. Blood samples were collected twice a week for HI antibody. The immunized raccoon dogs were euthanized after 14 days. **a** Images of injection site necropsy and tissue section captured by camera. **b** Changes in physiological parameters of immunized raccoon dogs after viral challenge. Groups of raccoon dogs (n = 5) were intramuscularly immunized with 10 µg, 50 µg, 100 µg VLPs treated with Al(OH)_3_, inactivated RDPV vaccine, PBS

Healthy Raccoon dogs were randomly divided into 5 groups and immunized with either different doses of RDPV VLPs (10 μg, 50 μg, 100 μg) treated with Al(OH)3 or 100 μL inactivated RDPV vaccine or PBS to examine whether RDPV VLPs could induce specific humoral immune responses. There were no sarcomas in the injection site after necropsy, which indicated that the absorption of the RDPV VLPs was good (Fig. 4a). There was a strong induction of High-titer HI antibodies which peaked (1:512) at 42 dpi, in the group receiving 100 μg RDPV VLPs as well as the vaccine group. In addition, a rise was seen in the humoral immune response. This was evident as a direct relation between an increase in HI antibody expression level in VLPs group with different doses of VLPs. In comparison with 100 μg group, the antibodies in the 10 μg and 50 μg groups did not increase significantly after day 14 post-inoculation. During the three-month immunization, however, the HI antibody titer remained above 1:80, which may be considered as conferring immune protection.

In order to determine whether the site of injection showed any pathological effects, the raccoon dogs were euthanized on day 14 after immunization. The site of injection was dissected and visually examined for the presence of any visible pathological signs such as the formation of nodules.

### Changes in the physiological parameters of immunized raccoon dogs upon virus challenge

**Fig. 5:**
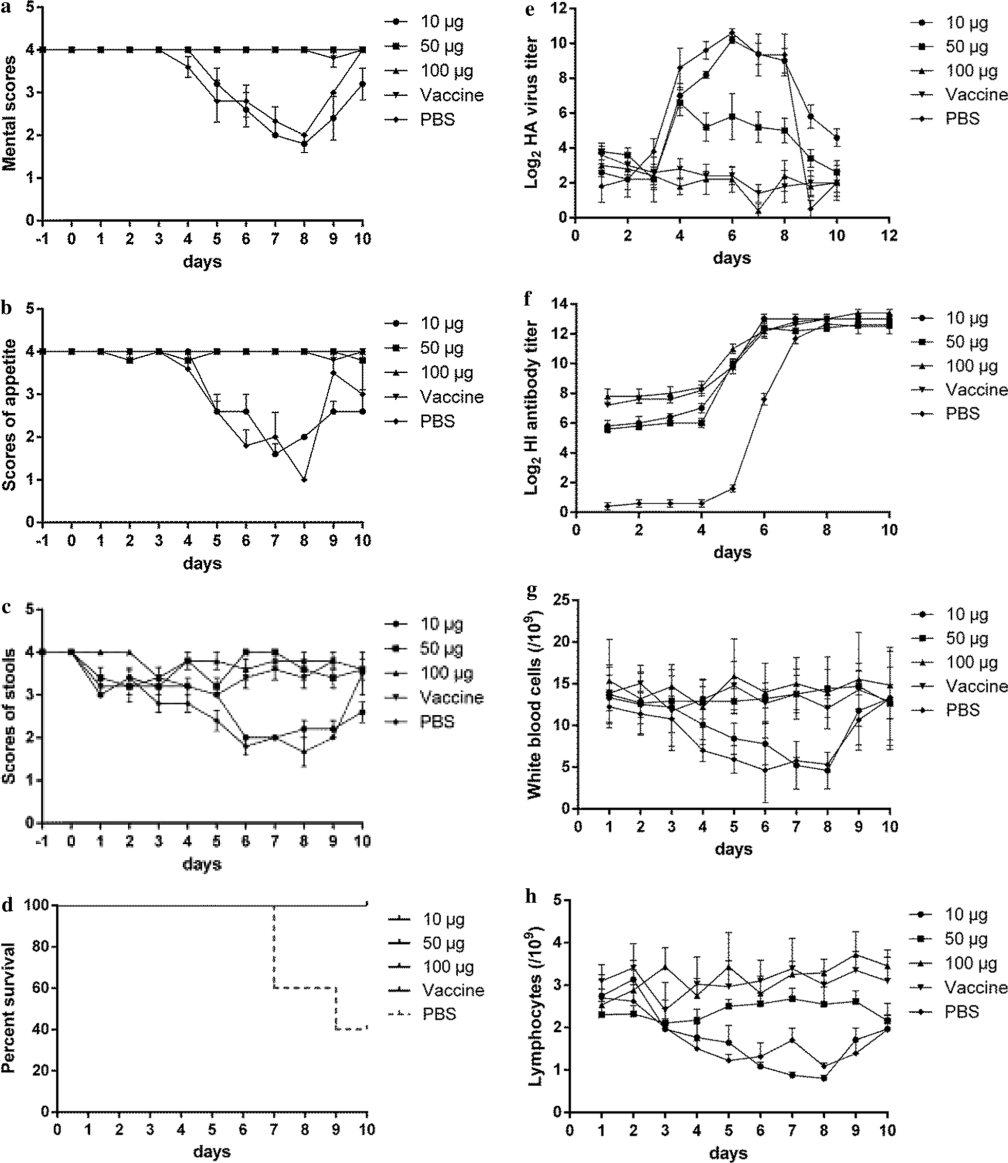
A summary of the clinical symptoms of the raccoon dogs and blood sample analysis. **a** Score for mental status of raccoon dogs. 1, Coma; 2, Listlessness; 3, Less energy; 4, Mentally normal. **b** The appetite status of the raccoon dogs. 1, Anorexia; 2, Loss of appetite; 3, Poor appetite; 4, Normal appetite. **c** Bowel movements of the raccoon dogs. 1, Hematochezia; 2, Watery stools; 3, Loose stools; 4, Normal stools. **d** Survival rate of raccoon dogs after oral administration of RDPV. **e** HA titer of stool samples. **f** HI titer of antibody in serum. **g** The number of white blood cells in the blood. **h** The number of lymphocytes in the blood

On day 21 after receiving a single dose of VLPs vaccine, the raccoon dogs were challenged orally with RDPV virus and the animals were observed for the development of any symptoms, which were recorded (Fig. 5). The animals in groups receiving 50 μg or 100 μg of Al(OH)_3_ treated RDVP VLPs or the vaccine group showed normal physical condition. However, animals in the group receiving groups 10 μg RDPV VLPs or receiving PBS showed the appearance of clinical symptoms related to RDPV approximately 4–5 dpi. There was 100% morbidity in the group receiving PBS. The clinical symptoms manifested were depression, diarrhea, and loss of appetite. These symptoms were most obvious in 7–8 days and began to gradually decrease after 9–10 days. Observation of the nasal cavity of sick raccoon dogs showed dryness along with general dehydration, dry skin and decreased elasticity. In addition, three of the five raccoon dogs of group PBS died (Fig. 5d). Almost all of the raccoon dogs began excreting the virus on the 4th day as detected using canine parvovirus detection kit. HA assay showed that the rates of viral excretion rose sharply on the 4th day (Fig. 5e). HI assay showed the antibodies in the serum of group 10 μg, 50 μg, 100 μg, vaccine began to rise sharply on the fourth day, while the antibody in the serum of group PBS only began to rise significantly on the 5th day (Fig. 5f). These results indicate that RDPV VLPs vaccine can protect raccoon dogs against RDPV RPSN infection, and 10 μg of RDPV VLPs is the lowest threshold for immune effectiveness. Changes in the number of white blood cells and lymphocytes also confirmed the effectiveness of the RDPV VLPs vaccine (Fig. 5g, h).

### H&E staining and immunohistochemical staining

**Fig. 6:**
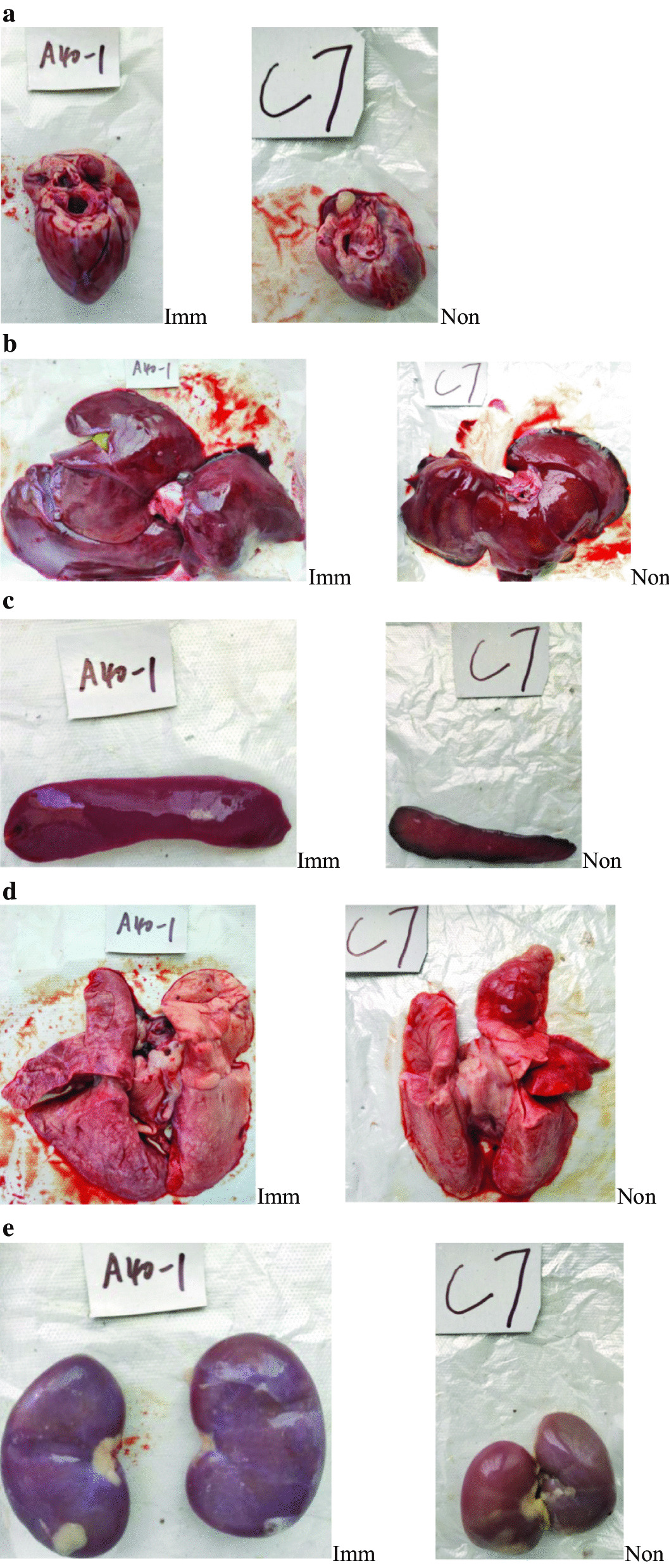

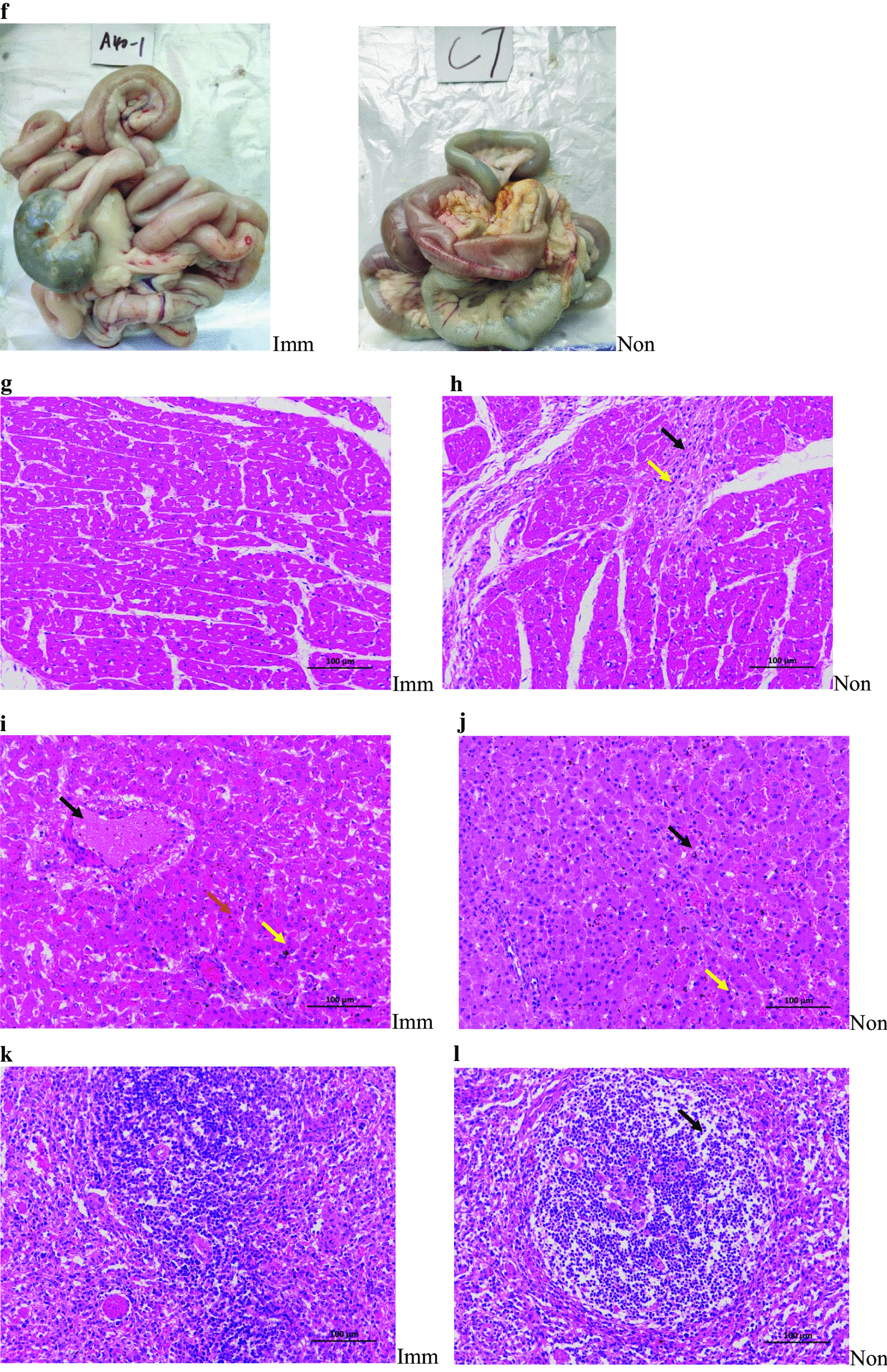

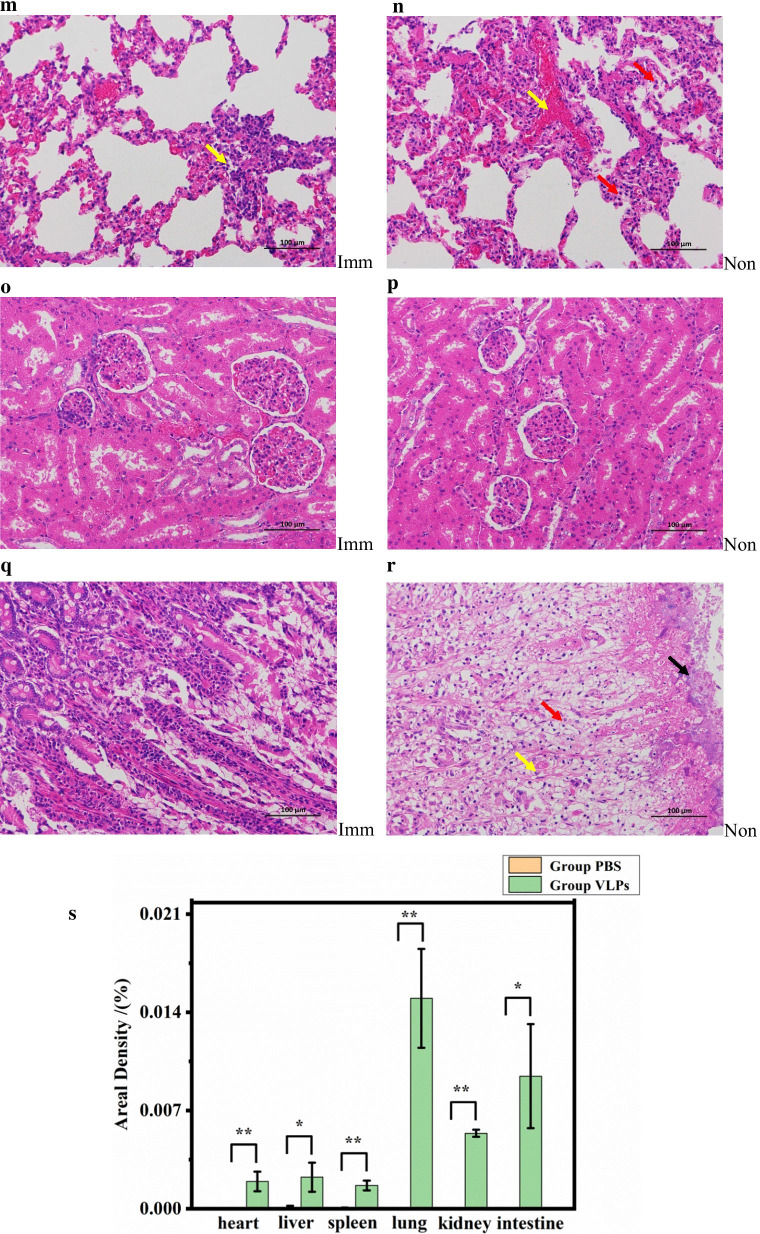
H&E staining and immunohistochemical staining (IHC) of organs from RDPV-inoculated raccoon dogs at 11 days. Non and Imm represent the non-immunized and immunized raccoon dog groups. **a** Heart. **b** Liver. **c** Spleen. **d** Lung. **e** Kidney. **f** Intestinal tract. **g** H&E staining of immunized raccoon dog heart. **h** H&E staining of non-immunized raccoon dog heart. A small amount of myocardial fibers can be seen in the visual field to dissolve, replaced by hyperplastic connective tissue (black arrow), accompanied by lymphocyte punctate infiltration (yellow arrow). **i** H&E staining of immunized raccoon dog liver. In the visual field, there is more congestion and dilation of liver sinusoids (black arrow), accompanied by a small amount of hemosiderin exudation (yellow arrow), local necrosis of hepatocytes and nuclear fragmentation (red arrow). **j** H&E staining of non-immunized raccoon dog liver. More hemosiderin can be seen in the visual field (black arrow), and a small amount of hepatocyte chromatin margins can be seen locally (yellow arrow). **k** H&E staining of immunized raccoon dog spleen. **l** H&E staining of non-immunized raccoon dog spleen. The number of lymphocytes in the spleen nodules is reduced, and the arrangement is loose (black arrow). A small number of macrophages are visible, and brown particles are visible in the cytoplasm of macrophages (yellow arrows). **m**, H&E staining of immunized raccoon dog lung. There is a small amount of lymphocyte infiltration in the lung (yellow arrows). **n** H&E staining of non-immunized raccoon dog lung. The lungs are accompanied by a small amount of lymphocytes and macrophages infiltration (red arrow). Venous congestion and expansion can be seen (yellow arrow). **o** H&E staining of immunized raccoon dog kidney. **p** H&E staining of non-immunized raccoon dog kidney. **q** H&E staining of immunized raccoon dog intestinal tract. **r** H&E staining of non-immunized raccoon dog intestinal tract. A large area of necrosis and lysis can be seen in the mucosal layer (black arrow), which is replaced by hyperplastic connective tissue (yellow arrow), accompanied by diffuse lymphocyte and macrophage infiltration (red arrow). **s** Analysis of positive areal density after immunohistochemical staining of organs in group VLPs and group PBS infected with RDPV RPSN. (rabbit anti-RPSN pAb, × 200, data are shown as mean ± SEM. The one-way ANOVA showed a statistically significant difference between group VLPs and group PBS. Single asterisk indicates statistical significance at *p* < 0.05, and double asterisk indicates *p* < 0.01

The dead raccoon dogs in group receiving PBS were dissected, and their organs were removed for further study. Animals in of group receiving 100 μg RDPV VLPs were euthanized for subsequent necropsy on day 11. Comparison of these two groups, changes in the physiological parameters of immunized raccoon dogs. Changes in the physiological parameters of immunized raccoon dogs upon virus challenge showed severe lesions except for the heart and kidneys (Fig. 6a, e). The results showed that RDPV RPSN may cause bleeding and necrosis in the liver and spleen, and at the same time, could also lead to enteritis (Fig. 6b, c, f). Additionally, the lobes of lungs were reddened and swollen, which may be due to hemorrhage in the surrounding lung (Fig. 6d). The H&E analysis of kidneys showed no microscopic lesions (Fig. 6o, p). However, other organs and tissues show several degrees of microscopic lesions. Of those, serious lesions were found throughout the intestinal tract. IHC evaluation was done for various organs (heart, liver, spleen, lung, kidney and intestinal tract) to check for the presence of virus in the tissues. The area density results from IHC showed proliferation of virus in tissues of heart, liver, spleen, lung, kidney, and intestinal tract in the group receiving PBS, while the tissues of the group receiving 100 μg VLPs showed almost no infection. The results also confirmed that IHC was able to identify parvovirus infection in tissues. These results also indicated that immunized animals were protected against tissue damage induced by RDPV RPSN.

## Discussion

At present, the inactivated vaccine is the only strategy available to fight RDPV disease. However, the major drawback of this approach is the use of formaldehyde as an inactivating agent. It is found to be toxic to raccoon dogs and causes local inflammation at the site of inoculation, which in some cases may lead to suppuration, and ulceration [6, 7]. A recent approach has been the development of VLP vaccines which has received widespread attention. VLPs mimic virus particles and present considerable epitopes and are, therefore, capable of stimulating multiple immune responses [17]. Previous studies have shown that a variety of VLPs protein could be expressed in various expression systems, such as mammalian, insects, yeast, and *E. coli* [4, 12, 14, 16, 20, 29]. To the best of our knowledge, there are no reports on the expression of RDPV VP2 till date. Alignment studies of RDPV VP2 genes and CPV VP2 genes have shown 99.9% identity at the nucleotide level, which indicated a very high degree of sequence homology [30]. Previous researchers have successfully reported the co-expression of CPV VP2 with Tf16 in *E. coli* [26]. Working on similar lines, an attempt was made to co-express RDPV VLPs and Tf16 in *E. coli*. The soluble RDPV VP2 expressed was found to induce a strong immune response in raccoon dogs. This is one of the pioneer reports studying the expression of RDPV VP2, and also evaluating the protective effect of drugs through HE staining and IHC.

*Escherichia coli* has significant advantages in expressing recombinant proteins, such as high expression and low cost. However, due to the highly toxic effects of *E. coli* endotoxin, contamination of the vaccination with it may cause body temperature to rise, disturb metabolic function, trigger the coagulation cascade and induce shock. Therefore, complete removal of endotoxin is a crucial step in the manufacturing process [23, 25]. Preliminary studies had suggested several methods for endotoxin removal such as the use of an organic solvent (1-octanol), CsCl density gradient centrifugation, anion exchange chromatography, detergent extraction (Triton X-100, Triton X-114), nickel affinity column chromatography, and use of Polymyxin B-immobilized cartridge [3, 8, 22, 31]. Considering the need for large-scale production of RDPV VP2 protein in the future, we attempted to standardize a new, efficient and cost-effective method for endotoxin removal. Following a single-cycle Triton X-114 extraction, the concentration of endotoxin in RDPV VP2 protein was reduced by more than 92%. It is being suggested that subsequent phase extraction cycles would further reduce the contamination with endotoxin (Table 1). These data are consistent with previous reports which have shown the efficacy of Triton X-114 extraction to reduce endotoxin concentration in expressed VP2 protein [28].

Per the previous reports that state that VP2 protein could be purified by density gradient ultracentrifugation [13, 19], a similar approach has been taken to purify the VLPs in our study. Hydrophobic chromatography used to purify VLPs has shown better recovery *vis* density gradient ultracentrifugation. Salt concentration and pH are known factors that affect the formation and stability of VLP [21]. Our experiments also indicated that RDPV VP2 protein could self-assemble to VLPs at pH 8.0 and 250 mM NaCl, which had a similar size, shape and a high HA titer (1:2^18^) to RDPV.

To assess the immunogenicity of RDPV VLPs, these were inoculated intramuscularly to the raccoon dogs. In contrast to other studies which have reported high titers of HI antibodies after two doses of immunization [15, 26], we observed the presence of high titers of HI antibody (1:4096) against RDPV after a single injection of 100 μg VLP. An immune response was observed in all the groups in groups receiving either 10 μg, 50 μg, 100 μg RDPV VLP or inactivated vaccine. No side-effects were observed in any of the groups as compared to controls, indicating the safety of RDPV VLP vaccine and implying that the above preparation process may be used for large-scale preparation of RDPV VP2. Further, the animals were challenged with the virus to evaluate the immune protection offered by the VLP vaccine.

The mechanism of VLP vaccine in inducing proliferation of CD4 + T cells and CD8 + T cells in mice has been well documented [9, 29]. VLP vaccines are recognized and engulfed by antigen-presenting cells in a manner similar to that of a viral infection. The vaccine can also directly stimulate dendritic cells, promote dendritic cells to produce pro-inflammatory factors, and induce cellular immunity [11]. Studies have also extensively documented that VLPs mainly stimulate T-helper type 1 (Th1) response in guinea pigs [15, 26]. Owing to these consistencies, similar analyses were not attempted in the present work.

We have found that the incubation period of RDPV is approximately 4 days, the clinical symptoms are most obvious in 7–8 days, and the symptoms begin to weaken gradually in 9–10 days. Listlessness, loss of appetite, loose stools are the common symptoms of the struck raccoon dogs. HA test reached detectable titers in 10 μg, PBS after 4 days. CPV test strips indicated that raccoon dogs could excrete virus through the stools after 4 days. A complete blood count showed a significant reduction in the numbers of both white blood cells and lymphocytes in the raccoon dog. This is in accordance with the recent study which has reported the decrease in WBC count upon challenge with RDPV [7]. We found a rapid increase in the HI antibody titers upon challenge test, which may indicate the involvement of memory B cells. Interestingly, the raccoon dogs in the group with 10 μg RDPV not only showed clinical symptoms and white blood cell changes, but also better survival as compared to control. Cumulatively, these results indicated that 10 μg of RDPV VLPs may be the lowest threshold for immune effectiveness.

Study shows that CPV causes hemorrhagic gastroenteritis in adult dogs and myocarditis in puppies [2]. We found the presence of lesions in various tissues including heart, lungs, liver, spleen and intestinal tract as examined by H&E. RDPV RPSN mainly targeting the intestinal tract. IHC results confirmed this as RDPV RPSN could be detected in 6 types of tissue, suggesting that RDPV RPSN was not only present but also able to replicate in these tissues. This suggests that RDPV was a pantropic virus, which could replicate in heart, liver, spleen, lung, kidney, intestinal tract. Since H&E staining can detect histological lesions, but may not detect parvovirus infection, thus, a combination of H&E staining and IHC can more clearly identify a parvovirus infection. These results are also consistent with findings in another study [18]. Sick raccoon dogs having obvious clinical symptoms show damage to multiple organs in a short period of time, and the mortality rate is extremely high. Therefore, the study needs to be replicated with a similar design, focusing on the identification of parvovirus infection.

In a nutshell, we have attempted to develop an effective method for preparing RDPV VP2 protein, which includes co-expression with Tf16 in *E. coli*, followed by purification, endotoxin removal and self-assembly. RDPV VP2 can assemble into a VLP similar to natural RDPV. Animal experiments have shown that VLP can stimulate the long-term immune response of raccoon dogs to RDPV, and no obvious side effects have been observed in the safety test of raccoon dogs. In vivo experiments also showed that immunization with RDPV VLP significantly lowered blood viral load upon subsequent challenge by the virus. The RDPV VLPs vaccine caused a higher specific humoral immune response against RDPV, and 10 g RDPV VLPs would be the lowest threshold of immune effect. In addition, this research test also shows that H&E staining and IHC can more clearly recognize parvovirus infection. This study provides new insights into the pathogenesis and clinical characterization of RDPV. RDPV VLPs vaccine has the potential to be exploited as a new commercial vaccine candidate against RDPV infection. This study also verified that the RDPV VLPs expressed by *E. coli* system may become a safe, effective, and economic candidate vaccine.

## Conclusions

In this study, we expressed a high-yield, soluble RDPV VP2 protein in *E. coli* cells. A novel method to purify the protein and remove endotoxin was also established. The protein prepared by this process successfully self-assembled into VLP, which showed a high titer of HA activity. RDPV VLP vaccination afforded protection to raccoon dogs and prevented organ damage upon RDPV challenge. These results suggest that RDPV VLPs vaccine expressed by *E. coli* system was safe and effective, and may be exploited as a promising vaccine against RDPV infection.

## Data Availability

All relevant information is provided in this current manuscript.

## Abbreviations

FPV: feline parvovirus
CPE: cytopathic effect
VLPs: virus-like particles
TF: trigger factor
*E. coli*: *Escherichia coli*
TEM: transmission electron microscopy
DLS: dynamic light scattering
HA: hemagglutination
HI: hemagglutination inhibition
